# Does husband’s alcohol consumption increase the risk of domestic violence during the pregnancy and postpartum periods in Nepalese women?

**DOI:** 10.1186/s12889-020-10021-y

**Published:** 2021-01-04

**Authors:** Narayan Bhatta, Sawitri Assanangkornchai, Ishwari Rajbhandari

**Affiliations:** 1 Epidemiology Unit, Faculty of Medicine, Prince of Songkla University, Hat Yai, 90110 Thailand; 2 Department of Neurosurgery, National Neurological Referral Center, Bir Hospital, Kathmandu, 44600 Nepal

**Keywords:** Domestic violence, husband’s alcohol drinking behavior, Pregnancy and postpartum

## Abstract

**Background:**

Domestic violence against women during pregnancy and the postpartum period not only violates the human rights of women but also harms on the health of both mother and child. Domestic violence is entrenching in social norms, customs and structural factors against women in Nepal. The use of alcohol also exacerbates domestic violence. The objective of this study was to determine the association between domestic violence against women and husband’s drinking behavior across the periods of pregnancy and postpartum.

**Methods:**

This study was a cross-sectional study conducted in the antenatal care and postnatal care clinics of a government hospital in Kathmandu district. Among 660 women (aged 15–49), 165 women were consecutively recruited from each trimester of pregnancy and the postpartum period. Adjusted odds ratios (AOR) were computed from a multivariate logistic regression model to determine the association between domestic violence against women and the husband’s drinking behavior.

**Results:**

Women whose husbands drank alcohol were twice as likely to suffer from domestic violence, compared to those women whose husbands did not drink (AOR = 2.12; 95% CI: 1.4–3.2), independently of their socio-demographic status. Women suffered from domestic violence in each period of pregnancy and postpartum due to their husband’s drinking habits, but the most affected period was the second trimester of pregnancy. Among women who suffered from physical, psychological and sexual violence during the pregnancy and postpartum periods, 70.2, 67.9, and 64.2% respectively experienced violence due to their husband’s drinking habit. Other associated factors for domestic violence included the ethnic culture of Janjati ethnicity, illiteracy of the women, duration of marriage 2–5 years (compared to one year or less) and a husband who behaved in a controlling manner.

**Conclusions:**

Having a husband who has alcohol drinking behavior is an important risk factor for domestic violence against women in the pregnancy and postpartum periods. Screening of alcohol use in husbands will not prevent domestic violence but could lead to a referral to integrated treatment for alcohol and domestic violence treatment.

**Supplementary Information:**

The online version contains supplementary material available at 10.1186/s12889-020-10021-y.

## Background

Domestic violence (DV) is one form of violence which may be physical, sexual, or psychological and perpetrated by one person on another within the family environment. DV in the pregnancy and postpartum periods are a public health challenge worldwide, affecting not only the women but also the fetus and newborn baby [1, 2]. Pregnancy and delivery bring many physiological and psychosocial changes in women and if she suffers from DV then the condition became worse, which increases the risk for mental problems such as depression, anxiety, etc. [3] Furthermore, a meta-analysis reported that a higher prevalence of DV against women during their pregnancy period was seen in developing countries (28%) compared to developed countries (13%) [4].

Alcohol is consumed by billions of people worldwide [5]. Alcohol harms not only the drinkers but also the family and the surrounding environment [6]. Alcohol consumption as a direct cause of intimate partner violence (IPV) has often been contested [7]. Alcohol fluctuate mood and increase existing feelings of anger and frustration [8]. Domestic violence rises in the context of alcohol use [9]. Women whose husbands drank alcohol were twice as likely to experience DV compared to women with non-drinking husbands [10, 11]. A study from Nepal reported that 48% of women had experienced DV during pregnancy because of their husband’s drinking habits and women experienced DV from their husbands if they gave birth to a daughter [12, 13]. In some cultures of Nepal, the bride’s family has to give dowry to the bridegroom’s family during the marriage ceremony which is an extra economic burden for the bridal family so that parents prefer the male child. Some culture treats girl child as another’s property and son as a supportive pillar for old age. Limited studies have been conducted in Nepal neither of them has mentioned the DV due to the husband’s alcohol drinking behavior in each period of pregnancy and postpartum.

Thus, hope that our study findings will enhance the understanding of alcohol-related DV during the pregnancy and postpartum periods and ease planning and intervention programs for addressing alcohol-related DV. The aim of this study is to examine the association between DV against women and their husband’s alcohol drinking behavior during the pregnancy and postpartum periods.

## Methods

### Study design, and study site

A cross-sectional study has conducted in Paropakar Maternity and Women’s Hospital (PMWH), a central government hospital in Kathmandu district, Nepal.

### Study population

The study population comprised women in the pregnancy and postpartum periods, aged 15–49 years, and attending antenatal care (ANC) and postnatal care (PNC) clinics in PMWH. The participants were women in the first, second and third trimester and mother in less than ten weeks postpartum mothers.

### Sampling technique and sample size

This paper is a part of a large study to investigate the prevalence of DV in each period of pregnancy and postpartum. The sample size was, therefore, calculated to answer the primary objective of the large study, where 165 women were consecutively recruited from each trimester of the pregnancy and postpartum periods, that is, 660 women in total. Different women were recruited in each period and the repetition of the same participant in different periods was not included. This sample size was deemed to have adequate power to test the hypothesis of a difference of 20% in the prevalence of DV between women with and without a drinking husband.

### Data collection tools and technique

Written informed consent was taken individually from all participants after informed them about the study and ethical issues involved. Each woman was interviewed separately inside the ANC and PNC clinic. Face to face interview was done by well-trained nursing staff from 8th December 2017 to 10th February 2018 with pregnant women and postpartum mothers. The questionnaire of the study was developed by adapting the questionnaire from the WHO multi-country study on domestic violence against women (2005) (see Additional file 1). Before data collection, the English version of the questionnaire was translated by a bilingual translator into the Nepali language and back-translated into English by another bilingual translator. Any discrepancies were noted and corrected by the principal investigator. A pilot study was conducted among 66 non-sampled women to evaluate the clarity, readability and comprehensibility of the questionnaire and necessary changes were made. The internal consistency of the questionnaire, computed by Cronbach’s alpha, was 0.72.

### Study variables

DV during the pregnancy and postpartum periods was the main outcome variable. DV may be physical, psychological and/or sexual and was defined as follows:

- Physical violence**:** any physical actions that hurt the partner including slapping, beating, pushing, burning, or threatening by the use of weapons against the woman.
- Psychological violence**:** use of threatening or offensive language and embarrassing, menacing or threatening to hurt a close person of the woman.
- Sexual violence**:** any attempt by the husband to force his wife to have sexual intercourse when she was ill, tired or not interested.

A woman was considered experiencing domestic violence if she answered “yes” to any of these forms of violence during pregnancy and postpartum periods. The women were asked if this violence could occur because of her husband’s drinking and the response was yes/no.

Independent variables consisted of social-demographic characteristics of the woman and her husband (age, religion, ethnicity, education, and residence), marriage type, and duration of being a couple, and husband’s alcohol drinking behavior and controlling behavior. The drinking behavior of the husband was determined from the woman’s responses to a simple alcohol quantity-frequency questionnaire in the past 12 months. The choices for frequency ranged from “every day” to “less than once per month” while those for quantity, ranged from 1 glass (equivalent to a standard drink) to more than 6 glasses of alcohol per day.

A husband was deemed to be a drinker if the wife stated that her husband drank alcohol or other local liquor, such as Rakshi, Tongba, Chhyang, Marpha, etc., more than 14 drinks per week (the composite of frequency quantity) during her pregnancy or postpartum periods. The woman was also asked if she drank alcohol as well as the frequency and quantity during the pregnancy and postpartum periods. We didn’t categories husband drinking behavior into any drinking or problem drinking. We only categories husbands drinking behavior into drinker or non drinker.

Husband’s controlling behavior was defined as one or more of the following acts of the husband against his wife to control her or force her to do something against her will, including; trying to prevent her from contacting her friends; restrict her from contacting family or relatives; frequently accusing the wife of behaving annoyingly; gets angry or jealous if she talks to other men and expecting her to ask for permission to exit the home even for a health checkup. A woman was considered experiencing the husband controlling behavior if she answered “yes” to any of this above act of husband during pregnancy and postpartum periods [14].

Pregnancy and the postpartum period: The trimesters of pregnancy were defined as the periods of pregnancy, ranging from 0 to 3 months (1st trimester), 4–6 months (2nd trimester), 7–9 months (3rd trimester) respectively, while the postpartum period was defined as the period ranging from 6 to 10 weeks after delivery.

### Data management and analysis

Data were coded and double-entered using Epi-Data 3.1. All analyses were done in R program. General information about the women was presented using frequencies (percentage) or mean and standard deviation (SD) as appropriate. To identify the association between DV in pregnancy and the postpartum periods and all independent variables, univariate analysis was conducted using the Chi-square test and Fisher’s exact test as appropriate. A multivariate logistic regression model was used to identify independent associations among variables having a *p*-value of 0.2 or less from the univariate analysis. To identify the strength of association for each variable, adjusted odds ratios (AOR) and 95% confidence intervals (CI) were presented.

## Results

### Socio-demographic and behavioral characteristics of the study participants

We had categorized the women into two age groups; approximately two-thirds (65.3%) were under 26 years old. The religion of most women was Hindu (75.5%);almost half (46%) belonged to the Janjati ethnic group. Most (94%) women were literate and 21% were residing outside the Kathmandu valley. The mean (SD) duration of marriage was 4.3 (3.6) years and about 39% of women were married to their current husbands for 2–5 years. Approximately 10% of women reported drinking alcohol during their pregnancy and postpartum periods. The mean (SD) age of husband was 28.0 (5.2) years and 4.2% were illiterate. Ten percent of women reported that their husbands had an extramarital affair, and approximately 50% of husbands drank alcohol during her pregnancy and postpartum periods. One in four women reported that their husbands exerted a controlling behavior on them.

Table 1 shows the association between husband’s drinking behavior and the different variables related to women and their husbands. Across all women and husbands characteristic variables, higher percentages of DV were seen among women whose husbands drank alcohol. Comparing within each woman variable, the proportion of DV was higher among younger, non-Hindu, Janjati, illiterate, drinking women and those who lived outside the Kathmandu valley, regardless of the drinking status of husband. Among women with drinking husbands, love marriages and marriage duration of 2–5 years were associated with a higher proportion of DV, compared to arranged marriages and shorter durations. The family’s preference for a male child was also associated with a higher percentage of the DV. Regarding husband’s characteristics, a higher proportion of DV was seen in women with illiterate and unemployed husbands, those with household income below the poverty line, and those whose husbands had an extramarital affair and exerted a controlling behavior.

**Table 1:** Socio-demographic characteristics of women and associations with domestic violence due to husband’s drinking behavior

Socio-demographic variables	Total *N* = 660	Overall domestic violence	Overall *P* value	Husband alcohol drinking behavior			
				Drinker		Non-drinker	
				DV	Total	DV	Total
				*n* = 109	*n* = 327	*n* = 56	*n* = 333
Age of women			0.278				
< =25	431	114 (26.5)		75 (35.7)	210	39 (17.6)	221
25>	229	51 (22.3)		34 (29.1)	117	17 (15.2)	112
Religion			< 0.001				
Hindu	498	108 (21.7)		74 (30)	247	34 (13.5)	251
Other^a^	162	57 (35.2)		35 (43.8)	80	22 (26.8)	82
Ethnicity			< 0.001				
Bramin/chhetri/Newar	356	57 (16)		37 (23.7)	156	20 (10)	200
Janjati	304	108 (35.5)		72 (42.1)	171	36 (27.1)	133
Address of women			< 0.001				
within Kathmandu	520	108 (20.8)		70 (26.7)	262	38 (14.7)	258
Outside Kathmandu	140	57 (40.7)		39 (60)	65	18 (24)	75
Education			< 0.001				
Literate	622	140 (22.5)		94 (30.8)	305	46 (14.5)	317
Illiterate	38	25 (65.8)		15 (68.2)	22	10 (62.5)	16
Occupational status			0.916				
Employed	156	40 (25.6)		32 (37.2)	86	8 (11.4)	70
Housewife	504	125 (24.8)		77 (32)	241	48 (18.3)	263
Marriage type			0.092				
Arranged marriage	383	86 (22.5)		52 (31.1)	167	34 (15.7)	216
Love marriage	277	79 (28.5)		57 (35.6)	160	22 (18.8)	117
Marriage duration(years)			0.001				
< =1	198	31 (15.7)		17 (19.5)	87	14 (12.6)	111
2–5	256	77 (30.1)		53 (41.1)	129	24 (18.9)	127
> =6	206	57 (27.7)		39 (35.1)	111	18 (18.9)	95
Pregnancy intended			0.1				
Wanted	571	136 (23.8)		90 (31.9)	282	46 (15.9)	289
Unwanted	89	29 (32.6)		19 (42.2)	45	10 (22.7)	44
Preference of Child			0.003				
Daughter	172	28 (16.3)		19 (24.7)	77	9 (9.5)	95
Son	488	137 (28.1)		90 (36)	250	47 (19.7)	238
Women’s drinking habit			0.079				
No	597	143 (24)		90 (32.4)	278	53 (16.6)	319
Yes	63	22 (34.9)		19 (38.8)	49	3 (21.4)	14
Family type			0.211				
Nuclear family	475	112 (23.6)		80 (33.5)	239	32 (13.6)	236
Joint family	185	53 (28.6)		29 (33)	88	24 (24.7)	97
Age of Husband			1				
< =25	230	57 (24.8)		36 (33.6)	107	21 (17.1)	123
25>	430	108 (25.1)		73 (33.2)	220	35 (16.7)	210
Husband education			0.004				
Literate	632	151 (23.9)		101 (32.3)	313	50 (15.7)	319
Illiterate	28	14 (50)		8 (57.1)	14	6 (42.9)	14
Occupational status			0.412				
Employed	617	157 (25.4)		103 (33.2)	310	54 (17.6)	307
Unemployed	43	8 (18.6)		6 (35.3)	17	2 (7.7)	26
Extra marital affair			< 0.001				
No	594	136 (22.9)		82 (29.4)	279	54 (17.1)	315
Yes	66	29 (43.9)		27 (56.2)	48	2 (11.1)	18
Control behavior by husband			< 0.001				
No	507	90 (17.8)		53 (22.9)	231	37 (13.4)	276
Yes	153	75 (49)		56 (58.3)	96	19 (33.3)	57
Household income			0.001				
Above poverty line	378	76 (20.1)		49 (26.9)	182	27 (13.8)	196
below poverty line	282	89 (31.6)		60 (41.4)	145	29 (21.2)	137

*P*-value is for comparison between levels of each variables and domestic violence due to husband’s drinking behavior

Numbers of frequencies and percentages in brackets were shown above

^a^Buddhist, Muslim, Christian, Kirat

### Association between different types of domestic violence and husband’s alcohol drinking behavior

Among women who experienced psychological violence during her pregnancy and postpartum periods, 67.9% reported that the violence was due to their husband consuming alcohol. Similarly, 70.2 and 64.2% of women who experienced physical and sexual violence reported that the violence occurred because of their husband drinking. Among all cases of violence, 66% were due to the husband’s alcohol drinking behavior. As shown in Table 2, there was a highly significant difference between different types of DV and husband’s alcohol consumption.

**Table 2:** Frequencies of different types of domestic violence and association with husband’s alcohol drinking behavior

Variables	Psychological violence	Physical violence	Sexual violence	Overall violence
	*n* = 84	*n* = 57	*n* = 106	*n* = 165
Husband drinking behavior				
No (*n* = 333)	27 (32.1%)	17 (29.8%)	38 (35.8%)	56 (33.9%)
Yes (*n* = 327)	57 (67.9%)***	40 (70.2%)**	68 (64.2%)***	109 (66.1%)***

P-value is for the comparison between husband’s drinking behavior and different types of violence * *p* < .05, ** *p* < .01, *** *p* < .001

Numbers of frequencies and percentage inside parentheses

Furthermore, among 10% of the women who reported drinking alcohol during their pregnancy and postpartum, 35% were suffering from DV. In the cases of both husband and wife drinking alcohol, 39% of women suffered from DV whereas only 32% reported DV if only husbands had a drinking habit.

### Prevalence of different types of domestic violence by period and husband’s alcohol drinking behavior

Table 3 shows a comparison of prevalence rates for each type of DV during the pregnancy and postpartum periods by the husband’s drinking status. Among all periods of pregnancy and the postpartum, women with drinking husbands had higher rates of each type of DV compared to those with a non-drinking husband. The highest percentage of DV among those with a drinking husband was seen in the second trimester of pregnancy (38.9%) while the rates in the first and third trimester of pregnancy and postpartum period were 26.6, 37.8, and 28.9% respectively. The rate of psychological violence was highest in the postpartum period (22.4%) while the rates of physical (17.8%) and sexual (31.7%) violence were highest in the second and third trimesters of pregnancy, respectively.

**Table 3:** Prevalence of different types of domestic violence by period of pregnancy and husband’s alcohol drinking behavior

	Pregnancy period						Postpartum period	
	1st trimester		2nd trimester		3rd trimester			
	Husband drinks		Husband drinks		Husband drinks		Husband drinks	
Types of Violence	Yes(*n* = 79)	No(*n* = 86)	Yes(*n* = 90)	No(*n* = 75)	Yes(*n* = 82)	No(*n* = 83)	Yes(*n* = 76)	No(*n* = 89)
Psychological	12.7%	4.7%	18.9%	13.3%	15.9%	9.6%	22.4%	5.6%
Physical	12.7%	1.2%	17.8%	12%	9.8%	7.2%	7.9%	1.1%
Sexual	16.5%	5.8%	22.2%	22.7%	31.7%	15.7%	11.8%	3.4%
Any violence	26.6%	9.3%	38.9%	28.0%	37.8%	22.9%	28.9%	9.0%

### Multivariate analysis

After bivariate analysis, significant variables remaining in the final multivariate logistic regression model are shown in Table 4. The husband’s drinking and controlling behavior, woman’s ethnicity, and educational status, and the duration of the marriage, was significantly and independently associated with DV. A woman who was married to a husband who drank alcohol had higher odds of suffering from DV. Additionally, women whose husbands exerted a controlling behavior on them were five times more likely to suffer from DV. Women from the Janjati ethnic group were twice more likely to suffer from DV than women from other ethnic groups and women who were illiterate were six times more likely to experience DV than literate women. Finally, women who had been married for 2 to 5 years were two times more likely to experience DV than women married for only one year or less.

**Table 4:** Crude and adjusted odds ratios (OR) of domestic violence with the characteristics of women and their husband

Variables	Crude OR (95%CI)	Adjusted OR (95%CI)	*P* value
Husband drink alcohol			< 0.001
No	1	1	
Yes	2.47 (1.7, 3.6)	2.12 (1.4, 3.2)	
Ethnicity of women			< 0.001
Bramin/chhetri/Newar	1	1	
Janjati	2.89 (2.0, 4.2)	2.43 (1.6, 3.7)	
Education of women			< 0.001
Literate	1	1	
Illiterate	6.62 (3.3,13.3)	6.09 (2.9–13)	
Marriage duration (years)			0.006
≤ 1	1	1	
2–5	2.32 (1.45,3.7)	2.27 (1.4, 3.8)	
≥ 6	2.06 (1.26,3.36)	1.68 (0.9, 2.9)	
Husband controlling behavior			< 0.001
No	1	1	
Yes	4.46 (3.02,6.58)	4.38 (2.9, 6.7)	

*OR* odds ratio, *CI* confidence interval

## Discussions

Pregnancy and the postpartum periods are a time of great vulnerability for some adverse consequences. Domestic violence occurring during this period can contribute to severe consequences. Many factors which are responsible for DV, but the alcohol drinking behavior of husband is one of the important factors for DV in Nepalese women [15]. Alcohol is culturally accepted in different ethnic groups in Nepal, especially in Newar and Janajati (alcohol drinking caste such as Tharu, Tamang) and used in most religious occasions [16]. A study conducted among 15–60 years men in Nepal found a high alcohol dependence prevalence (26%) [17]. Alcohol drinking behavior can lead to hostility and DV in case of an interpersonal clash. Alcohol drinking can impair cognitive and physical functions, which in turn reduce self-control and leaves individuals less capable of negotiating a non-violent resolution of conflicts within relationships [18, 19].

Alcohol works indirectly as a mood enhancer, which can increase the existing feelings of anger and frustration during the DV period [8]. Strong links have been found between alcohol use and the occurrence of DV [19]. Our study showed that women whose husbands had alcohol drinking behavior were two times more likely to experience DV during pregnancy, a result consistent with studies from other countries [20, 21]. The reason for this may be that the husband’s alcohol consumption habit may decrease the threshold of violence, but increase his negligence of household duties [22]. Furthermore, some husbands may use alcohol intentionally to behave violently against their partners [23].

To the best of our knowledge, this study is the first initiation to investigate the association between alcohol-related DV in each trimester of pregnancy and the postpartum period in Nepal. This study shows that women whose husbands drink alcohol had a higher risk of suffering from DV than women whose husbands did not drink. Women were suffering from DV in all four periods; the most affected period being the second trimester of pregnancy, a result consistent with a study conducted in Sweden [24]. Among women whose husbands drank alcohol, 38.9% experienced DV in their second trimester of pregnancy. The reason may be that, in the second trimester of pregnancy, there are visible structural changes in a woman’s body, making them not able to fulfill the psychosexual need of the husband and this contributes to the violent behavior of the husband against the woman. Another reason may be that women were much more aware of their pregnancy in the second trimester, so they tried to limit household activities as well as sexual activities with their husbands, a behavior that may have upset their husbands and subsequently leads to DV. Our study shows that all husbands having drinking behavior are not violent and not all violent partners have drinking behavior. Thus, the surrounding environment motivates the husband to continue drinking behavior which may be important for IPV during pregnancy and the postpartum period and it will be useful for future research [25].

Among all women who experienced DV in their pregnancy and postpartum periods, 66% reported that DV was associated with their husband’s alcohol drinking behavior. Consistent with our findings, women reported suffering from DV during pregnancy due to their husband’s drinking behavior in different studies conducted in India [26, 27]. In Nepal, drinking alcohol is a normal due to cultural activities in most of the ethnic groups. When women are not able to complete their household tasks during pregnancy or the postpartum period, it leads to quarrels with their husband that is often exacerbated by alcohol use.

Physical violence was the most prevalent violence reported by women. Of the women who experienced physical violence, 70.2% were associated with the alcohol drinking behavior of the husband. A similar finding has revealed in the Ethiopian study, which mentioned that among pregnant women who were suffered from physical violence, 44.83% women experienced physical violence due to husband drinking behavior [28]. Other studies; done in Brazil [29] and Thailand [30] revealed that women were more likely to experience domestic violence if husband’s consume alcohol. The reason for this may be that women made frequent attempts to cope with the drinking habit of their husband, but were unable to maintain their marital relationship because of physiological changes in their body.

In this study, the husband was the sole perpetrator of all types of DV during pregnancy and postpartum period. Consistent result have shown in other study [31] that the husband was the only perpetrator of physical, psychological and sexual violence. Other studies reported that other family members such as the mother-in-law, sister-in-law and father-in-law were also perpetrators of domestic violence [32, 33].

In Nepal, alcoholism is a common cause of familial conflict. The alcohol drinking behavior of the husband is the risk factor of DV during pregnancy and the postpartum period in Nepalese women [14, 15]. However, DV during the pregnancy and the postpartum periods can be affected by other factors such as ethnicity [34] and educational status of women, marriage duration [34] and husband controlling behaviors [14]. The male identity and the traditional masculine gender norms, as well as unequal power between male and female undeniably linked with DV where the use of alcohol aggravates the DV [35].

Alcohol also causes the unequal power relations and male-dominant gender roles by promoting them towards gender transformative behavior [35].

## Limitations

Our study has some limitations which should be acknowledged. Firstly, the cross-sectional study design precludes us from making causal influences. Secondly, because of limited data on severity of domestic violence and quantity and frequency of alcohol consumption of the husband, a dose-response relationship between these two variables could not be calculated. Thirdly, the study was institutional based, so we cannot generalize the results to all pregnant women in Nepal. Fourthly, the study may has suffered from response bias due to the highly sensitive issues inherent in the topic of domestic violence. Besides, husband’s drinking behavior was only asked from the woman, not directly from the husband, only approximated information was obtained and depending on the woman’s perception of her husband’s drinking. Finally, our study was conducted in women during their pregnancy and postpartum period in the Nepalese setting; it may not be applicable to another country setting.

## Conclusions

This study shows a high prevalence of domestic violence against women and that alcohol consumption is a significantly associated reason for DV during the pregnancy and postpartum periods. The results suggest that a proper screening program during antenatal and postpartum clinic visits may play a crucial role in uncovering hidden cases of DV and prevent them from further adverse health effect. The study also identifies the husband as the main perpetrator for DV. There have been several successful strategies for tackling domestic violence and harmful use of alcohol in general reported in the literature, such as reducing alcohol availability [36]. Such strategies should take into account the issues of social tolerance towards domestic violence, acceptance of excessive drinking as a mitigating factor, and normative beliefs about masculinity and heavy drinking. In addition, the higher vulnerability to physiological and psychological stresses of the women in pregnancy and postpartum periods should be considered in planning a specific intervention to prevent alcohol-related domestic violence among pregnant women and postpartum mothers. Alcohol-related intervention programs such as alcohol abuse awareness programs for both husband and wife and women’s empowerment programs directed to specific target groups may also help reduce the risk of DV in Nepal. Further research is also needed to identify the mechanism by which husband alcohol drinking contributes to the severity of violence during pregnancy and the postpartum period**.**

## Supplementary Information

**Additional file 1.** Study questionnaire. There were two types of questionnaire used for data collection. One questionnaire was for pregnant women, and another one for postpartum period mother.

## Data Availability

All data based on our study are available. As further studies will be conducted based on these data, we will thus not able to share the data but may possibly be available from the corresponding author on reasonable request.

## Abbreviations

DV: Domestic violence
WHO: World Health Organization
PMWH: Paroparkar Maternity and Women’s Hospital
ANC: Antenatal care
PNC: Postnatal care
OR: Odds ratio
IPV: Intimate partner violence
