# Supplementary material for: Does husband’s alcohol consumption increase the risk of domestic violence during the pregnancy and postpartum periods in Nepalese women?
Source: BMC Public Health. 2021 Jan 4;21:5. doi: 10.1186/s12889-020-10021-y (PMC7780634; doi:10.1186/s12889-020-10021-y)
Supplement: Supplementary file 1 — Additional file 1. Study questionnaire. There were two types of questionnaire used for data collection. One questionnaire was for pregnant women, and another one for postpartum period mother. [file 12889_2020_10021_MOESM1_ESM.zip › English version for pregnant womenR11.pdf]

Questionnaire for pregnant women

Pattern of domestic violence in women over the period of pregnancy and Post-partum

CONFIDENTIALITY: All information obtained about any individual respondent will be held strictly confidential

IDENTIFICATION

ID: [ ] [ ] [ ] [ ] [ ] [ ]

Date: / /

Name of interviewer: ________________________________

Hospital: 1. Paropakar maternal and women's hospital

S.N	**Section 1: Socio demographic factors**	Code
1.	What is your age? [write in Years]	agew ▢▢
2.	What is your Religion?<br>1.Hindu 3. Christian 5. Other…….<br>2.Buddhism 4. Muslim	rel ▢
3.	What is your Ethnicity?<br>1. Brahmin 3. Newar 5. Other…………<br>2. Chhetri 4. Tharu	ethn ▢
4.	What is your Current address?<br>1. Within Kathmandu 2. Outside Kathmandu	add ▢
5.	What is your education status?<br>1. Illiterate 4. Higher secondary level<br>2. Primary 5. University level<br>3. Secondary level	edn ▢
6.	What is your occupation status?<br>1. Unemployed 4. Government employee<br>2. Farmer 5. Housewife<br>3. Laborer 6. Business 7. Other…………	occu ▢
7.	Household income/month Rs……………………	hhI ▢

8.	Marriage type<br>1. Love marriage 2. Arranged marriage	mart ▢
9.	How many years you are in married relationship?<br>………years	mard ▢
10.	What is your Marital status?<br>1. Married 2. Divorced<br>3. Separated 4. Widowed	mars ▢
11.	Did your marriage involve dowry/bride price payment?<br>Yes<br>No skip Q 12,13	mardow ▢
12.	Has all of the dowry/bride price been paid for or does some part still remain to be paid?<br>1. All paid<br>2. Partially paid<br>3. None paid	dowp ▢
13.	Overall, do you think that the amount of dowry/bride price payment has had a positive impact on how you are treated by your husband and his family, a negative impact or no particular impact?<br>1. Positive impact<br>2. Negative impact<br>3. No impact	impdow ▢
	**Section 2 Maternal health**	
14.	What is your gestational age?<br>…………………. months	gesage ▢
15.	How many times you have antenatal care (ANC) visit<br>1. 1st time 2. 2nd times<br>3. 3rd or more times 4. Never	anct ▢
16.	Did your husband stop you, encourage you or have no interest in whether you received antenatal care for your pregnancy?<br>1.Stop you<br>2.Encourage<br>3.No interest	husenc ▢
17.	Thinking back to just before you got pregnant with your new baby, how did you feel about becoming pregnant?<br>1. I wanted to be pregnant<br>2. I didn’t want to be pregnant<br>3. I wasn’t sure what I wanted	pregwan ▢

18.	Have you ever been pregnant before-including pregnancies that did not end in a live birth, miscarriage or still birth or abort?<br><br>1.yes<br>2. No<br><br>if yes<br>1. Miscarriage (Pregnancy that ends on its own, within the first 20 weeks of gestation)<br>2. Abortions (termination of a human pregnancy during the first 28 weeks of pregnancy)<br>3. Still births (When a baby dies in utero at 20 weeks of pregnancy or later)<br>4. Antepartum hemorrhage (Vaginal bleeding during pregnancy from the 28th week of gestational age to term.<br>5. Other	hpregp<br><br>pregc<br><br>misc<br>abo<br>stb<br>aph<br>othp
19.	How old were you when you got the 1st pregnancy?<br>……yrs	firprega
20.	Number of children<br>Male 1.one child 2. Two children 3. Three or more 4.No<br>Female 1.one child 2. Two children 3. Three or more 4.No	malec<br>femc
21.	When you are pregnant, with this child, did your husband have preference for a son, a daughter or did it not matter to him whether it was a boy or a girl?<br>1. Son<br>2. Daughter<br>3. Did not matter	prefc
22.	Weight gain in pregnancy…..kg	weight
23.	During your current/most recent pregnancy, did you have any of the following health conditions?<br>For each one, check No if you did not have the condition or Yes if you did.<br>Yes No<br>a. Gestational diabetes (started during this pregnancy)<br>b. High blood pressure (started during this pregnancy)<br>c. Depression (feelings of severe despondency and dejection)<br>d. Abortion (termination of a human pregnancy during the first 28 weeks of pregnancy)<br>e. Other………………………	help

24.	Do you drink alcohol?<br>Yes ▢<br>No ▢ if no skip Q 25	dria▢
25.	How often do you drink alcohol?<br>1. Every day<br>2. Once or twice a week<br>3.1-3 times a month<br>4. less than once a month	drid▢
	**Section 3: Husband related factors**	
26.	How old is your husband?<br>Age …..yrs	hage ▢
27.	Education status of husband<br>1. Illiterate level 4. Higher secondary level<br>2. Primary 5. University level<br>3. Secondary level	hedn ▢
28.	Occupation status of husband<br>1. Unemployed 4. Government employee<br>2. Farmer 5. business<br>3. Laborer 6. Other…………	hoccu ▢
29.	What is your husband Religion?<br>1. Hindu 3. Christian 5. Other…….<br>2. Buddhism 4. Muslim	hrel▢
30.	What is your husband Ethnicity?<br>1. Brahmin 3. Newar 5. Other………….<br>2. Chhetri 4. Tharu	hethn▢
31.	Do your husband drink alcohol?<br>1. Yes<br>2. No If no skip Q 32-33	hdria ▢
32.	How often do your husband drink alcohol?<br>1. Every day 2. Once or twice a week<br>3.1-3 times a month 4. Less than once a month	hdriad ▢
33.	On the days that your husband drank, about how many alcoholic drink did he usually have a day?<br>1. One glass 3. 4-6 glasses<br>2. two to three glass 4. > 6 glasses	driaa▢
34.	Does your husband have another wife or girlfriend?<br>1.Yes ▢<br>2.No ▢<br>3. Don't know ▢	anowng▢

Section 4: Violence related questions		
When two people marry or live together, they usually share both good and bad moments. I would now like to ask some questions about your current and past relationships and how your husband treats you. If anyone interrupts us I will change the topic of conversation. I would again like to assure you that your answers will be kept secret, and that you do not have to answer any questions that you do not want to. May I continue?		
35.	In general, do you and your husband discuss the following topics together<br>Yes No<br>1. Things that have happened to him in a day ☐ ☐<br>2. Things that have happened to you in a day ☐ ☐<br>3. Your worries or feeling ☐ ☐<br>4. His worries or feeling ☐ ☐	discus ☐
36.	In your relationship with your husband, how often would you say that you quarreled?<br>1. Rarely ☐ 3. Often ☐<br>2. Sometimes ☐ 4. Never ☐	quarrel ☐
37.	Do you know about Domestic violence?<br>Domestic violence is a pattern of behavior which involves violence or other abuse by one person against another in a domestic setting, such as Scold you, insulting you in front of other person, hit you, restrict you to do something, doing sexual activity without your permission<br>1. Yes ☐ 2. No ☐	dv ☐
38.	I am now going to ask you about some situations that are true for many women. Thinking about your husband, would you say it is generally true that he:<br>Yes No<br>1.Tries to keep you from seeing your friends ☐ ☐<br>2.Tries to restrict contact with your family of birth ☐ ☐<br>3.Insists on knowing where you are at all times ☐ ☐<br>4.Gets angry if you speak with another man ☐ ☐<br>5.Is often suspicious that you are unfaithful ☐ ☐<br>6.Expects you to ask his permission before seeking health care for yourself ☐ ☐	conb ☐<br>☐<br>☐<br>☐<br>☐<br>☐<br>☐
39.	The next questions are about things that happen to many women, and that your husband may have done the following things to you. I want you to tell me if your husband has ever done the following things to you during pregnancy.	psyv ☐

	Yes No<br>1. Insulted you or made to feel bad about yourself? ☐ ☐<br>2. Belittled or humiliated you in front of other people? ☐ ☐<br>3. Did things to scare or intimidate her on purpose (e.g. by yelling or smashing things) ☐ ☐<br>4. Threatened to hurt you or someone you cared about? ☐ ☐<br>If not skip Q 40,41		☐<br>☐<br>☐<br>☐
40.	How frequently it was happened during pregnancy	1. Never ☐ 5. Monthly ☐<br>2. Once ☐ 6. Weekly ☐<br>3. 2-5 times ☐ 7. Daily ☐<br>4. >5times and <12 times ☐	psyvf ☐
41.	Do you think that this thing(s) would happened if your husband was drinking?<br>1.Yes<br>2. No		psyva ☐
42.	Has this happened before pregnancy<br>Yes No<br>1. Insulted you or made to feel bad about yourself? ☐ ☐<br>2. Belittled or humiliated you in front of other people? ☐ ☐<br>3. Did things to scare or intimidate her on purpose (e.g. by yelling or smashing things)? ☐ ☐<br>4. Threatened to hurt you or someone you cared about? ☐ ☐		histpsy ☐<br>☐<br>☐<br>☐<br>☐
43.	Has your husband ever done during pregnancy?<br>Yes No<br>1. Slapping or thrown something at you to hurt you ☐ ☐<br>2. Pushed you or shoved you or pulled your hair ☐ ☐<br>3. Hit you with fist or something else that could hurt ☐ ☐<br>4. Choking or Burning you on purpose ☐ ☐<br>5. Kicked you or beaten you in abdomen ☐ ☐<br>6. Threatened to use or actually used a weapon against you ☐ ☐<br>If not skip to 44 and 45		phyv ☐<br>☐<br>☐<br>☐<br>☐<br>☐<br>☐
44.	How frequently it was happened during pregnancy	1. Never ☐ 5. Monthly ☐<br>2. Once ☐ 6. Weekly ☐<br>3. 2-5 times ☐ 7. Daily ☐<br>4. >5times and <12 times ☐	phyvf ☐

45.	Do you think that this thing(s) would happened if your husband was drinking?<br>1. Yes ▢ 2. No ▢	phyva ▢
46.	Physical violence by an intimate partner before pregnancy<br>Yes No<br>1. Slapping or thrown something at you to hurt you ▢ ▢<br>2. Pushed you or shoved you or pulled your hair ▢ ▢<br>3. Hit you with fist or something else that could hurt ▢ ▢<br>4. Choking or Burning you on purpose ▢ ▢<br>5. Kicked you, dragged you or beaten you ▢ ▢<br>6. Threatened to use or actually used a weapon against you ▢ ▢	histphyv ▢<br>▢<br>▢<br>▢<br>▢<br>▢<br>▢
47.	Has your husband ever done sexual violence during pregnancy?<br>Yes No<br>1. Physically forced to have sexual intercourse when you did not want to? ▢ ▢<br>2. Had sexual intercourse when you did not want to because you were afraid of what he might do? ▢ ▢<br>3. Did he forced you to do something sexual that you found degrading or humiliating? ▢ ▢<br>If no skip Q 48, 49	sexv ▢<br>▢<br>▢<br>▢
48.	How frequently it was happened during pregnancy | 1. Never ▢ 5. Monthly ▢<br>2. Once ▢ 6. Weekly ▢<br>3. 2-5 times ▢ 7. Daily ▢<br>4. >5times and <12 times ▢	sexvf ▢
49.	Do you think that this thing(s) would happened if your husband was drinking?<br>1. Yes 2. No	sexva ▢
50.	Family type<br>1. Nuclear family Skip Q 51-56<br>2. Joint family	famt ▢
51.	I am now going to ask you about some situations that are true for many women. Thinking about your mother in-law, would you say it is generally true that she:<br>Yes No<br>1. Tries to control nutritious foods because of food taboos ▢ ▢<br>2. Tries to restrict contact with your family of birth ▢ ▢<br>3. Insists on knowing where you are at all times ▢ ▢<br>4. Gets angry if you speak with another man ▢ ▢<br>5. Is often suspicious that you are unfaithful ▢ ▢<br>6. Expects you to ask his permission before seeking health care for yourself ▢ ▢	conb ▢<br>▢<br>▢<br>▢<br>▢<br>▢<br>▢

52.	This questions are about mother in-law care and violence. This thing that happen to many women, and that your mother-in law may have done the following things to you.<br>I want you to tell me if your mother in-law has ever done the following things to you during pregnancy.<br>Yes No<br>1. Insulted you or made to feel bad about yourself? ☐ ☐<br>2. Belittled or humiliated you in front of other people? ☐ ☐<br>3. Threatened to hurt you ☐ ☐	psyv ☐<br>☐<br>☐<br>☐
53.	Has this happened before pregnancy<br>Yes No<br>1. Insulted you or made to feel bad about yourself? ☐ ☐<br>2. Belittled or humiliated you in front of other people? ☐ ☐<br>3. Threatened to hurt you ☐ ☐	histpsy ☐<br>☐<br>☐<br>☐
54.	Has your mother-in-law ever done the following things to you during pregnancy?<br>Yes No<br>1. Slapping and thrown something at you to hurt you ☐ ☐<br>2. Increase work pressure if you not able to work ☐ ☐<br>3. Pushed or shoved you or pulled your hair ☐ ☐<br>4. Hit with fist or something else that could hurt ☐ ☐<br>5. Choked or burnt you on purpose? ☐ ☐	phyvm ☐<br>☐<br>☐<br>☐<br>☐<br>☐
55.	Now I am going to asking about any types of domestic violence caused by any other people in the family<br>1.Yes<br>2.No if no skip Q 56<br>1. Father-in-law ☐ 4. Any other family member ☐<br>2. Sister-in-law ☐ 5. Other ☐<br>3. Brother-in-law ☐	dvo ☐
56.	What types of domestic violence done by ………….?<br>1. Physical ☐ 3. Sexual ☐<br>2. Psychological ☐ 4. Economical ☐	dvt ☐
57.	In your opinion, does a man have a good reason to hit his wife if:<br>Yes No<br>a) if she does not complete her household work to his satisfaction ☐ ☐<br>b) if she disobeys him ☐ ☐<br>c) if she argues with him ☐ ☐<br>d) if she disrespects her in-laws ☐ ☐<br>e) if she goes out without permission ☐ ☐<br>f) if she refuses to have sex with him ☐ ☐<br>g) if he finds out that she has been unfaithful. ☐ ☐	opihit ☐<br>☐<br>☐<br>☐<br>☐<br>☐<br>☐

	The responses were categorized as not accepting to any of the above acts, partially accepting (1–3 acts) and highly accepting (4–7 acts).	
58.	In your opinion, can a married women refuse to have sex with her husband if: Yes No<br>1. She does not want to ▢ ▢<br>2. He is drunk ▢ ▢<br>3. She is sick ▢ ▢<br>4. He mistreats her ▢ ▢<br><br>The responses were categorized as: completely refuse (in all matters) and does not refuse at all or partially refuse (1–2matters).	opisex ▢<br>▢<br>▢<br>▢<br>▢
	**Section 5: Injuries**	
59.	Have you ever been injured as a result of violence/abuse by your husband or other family members?<br>Yes ▢<br>No ▢ If no Skip 60-63	vioinj ▢
60.	In your life time how many times were you injured by your husband or other family member<br>1. Once/twice ▢<br>2. Several (3-5) times ▢<br>3. Many (more than 5) times ▢	injd ▢
61.	What types of injuries did you have? Yes No<br>1. Cuts, punches, bites ▢ ▢<br>2. Scratch, abrasion, bruises ▢ ▢<br>3. Burns ▢ ▢<br>4. Penetrating injuries, deep cuts, gashes ▢ ▢<br>5. Broken ear drum, eye injuries ▢ ▢<br>6. Fractures, broken bones ▢ ▢<br>7. Other: ……………………….. ▢ ▢	injtyp ▢
62.	Did you ever receive health care or visited hospital for your injury?<br><br>1.Yes sometimes ▢<br>2. Yes always ▢<br>3. No ▢<br>Times……….	helcar ▢
63.	Did you tell a health worker the real cause of your injury?<br><br>1.Yes ▢ 2. No ▢	tellhel ▢

Section 6: Financial related factors		
64.	Does your husband ever refuse to give you money for household expenses or treatment,even when he has money for other things?<br><br>1. Never ☐<br>2. Once ot twice ☐<br>3. Several times ☐<br>4. N/A(partner doesnot earn money) ☐	husmon ☐
65.	Have you ever given up/ refused a job for money because your husband did not want you to work?<br>1.Yes ☐<br>2. No ☐	refjob ☐
66.	Have you ever face financial burden because of violence<br><br>1.Yes ☐<br>2.No ☐	finbv ☐

I would like to thank you very much for helping us. I appreciate the valuable time that you giving me and talking about your personal matters. I realize that these questionnaires may have been difficult for you to answer, but it is only by hearing from women themselves that we can really understand about their health and experiences of violence. However, from what you have told me I can see that you are strong and have survived thought some difficult circumstances.

Do you have any questions for me or would like to give any suggestions?

Thank you so much
